# Prolonged astrocyte-derived erythropoietin expression attenuates neuronal damage under hypothermic conditions

**DOI:** 10.1186/s12974-020-01831-3

**Published:** 2020-05-02

**Authors:** Kohki Toriuchi, Hiroki Kakita, Tetsuya Tamura, Satoru Takeshita, Yasumasa Yamada, Mineyoshi Aoyama

**Affiliations:** 1 Department of Pathobiology, Nagoya City University Graduate School of Pharmaceutical Sciences, 3-1 Tanabedori, Mizoho-ku, Nagoya, Aichi 467-8603 Japan; 2 Department of Perinatal and Neonatal Medicine, Aichi Medical University, 1-1 Yazakokarimata, Nagakute, Aichi 480-1195 Japan; 3 Department of Anesthesiology and Intensive Care Medicine, Nagoya City University Graduate School of Medical Sciences, 1 Kawasumi, Mizuho-ku, Nagoya, Aichi 467-8601 Japan

**Keywords:** Astrocytes, Erythropoietin, Hypothermia, Hypoxia, Neuroprotection

## Abstract

**Background:**

Hypoxic-ischemic encephalopathy (HIE) has a high morbidity rate and involves severe neurologic deficits, including cerebral palsy. Therapeutic hypothermia (TH) has been shown to decrease the mortality rate and provide neuroprotection in infants with HIE. However, death and disability rates in HIE infants treated with TH remain high. Although the cellular mechanism of the neuroprotective effect of TH remains unclear, astrocytic erythropoietin (EPO) is known to be a key mediator of neuroprotection under hypoxic conditions. In the present study, we investigated the hypothermia effect on EPO expression in astrocytes and determined whether hypothermia attenuates neuronal damage via EPO signaling.

**Methods:**

Astrocytes derived from rat cerebral cortex were cultured under oxygen/glucose deprivation (OGD). The expression of EPO and hypoxia-inducible factor (HIF), a transcription factor of EPO, was assessed. After OGD, astrocytes were cultured under normothermic (37 °C) or hypothermic (33.5 °C) conditions, and then EPO and HIF expression was assessed. After OGD, rat cortical neurons were cultured in astrocyte-conditioned medium (ACM) derived from the hypothermic group, and neuronal apoptosis was evaluated.

**Results:**

OGD induced EPO mRNA and protein expression, although at lower levels than hypoxia alone. HIF-1α and HIF-2α protein expression increased under hypoxia alone and OGD, although OGD increased HIF-2α protein expression less than hypoxia alone. EPO gene and protein expression after OGD was significantly higher under hypothermia. Moreover, expression of HIF-1α and HIF-2α protein was enhanced under hypothermia. In the presence of ACM derived from hypothermic astrocytes following OGD, the number of cleaved caspase 3 and TdT-mediated dUTP nick-end labeling-positive apoptotic neurons was lower than in the presence of ACM from normothermic astrocytes following OGD. Blockade of EPO signaling using anti-EPO neutralization antibody attenuated the anti-apoptotic effect of ACM derived from hypothermic astrocytes following OGD.

**Conclusions:**

Hypothermia after OGD stabilized HIF-EPO signaling in astrocytes, and upregulated EPO expression could suppress neuronal apoptosis. Investigating the neuroprotective effect of EPO from astrocytes under hypothermic conditions may contribute to the development of novel neuroprotection-based therapies for HIE.

## Background

Perinatal asphyxia-induced brain injury may present as hypoxic-ischemic encephalopathy (HIE) in the neonatal period [1]. The prevalence of HIE is 3 to 5 cases per 1000 live births, and the condition has a high morbidity rate and involves severe, long-term neurologic and cognitive deficits with high rates of cerebral palsy and mental retardation [1].

Treatment of HIE with hypothermia has become standard care for infants [2, 3]. Therapeutic hypothermia (TH) has been shown to decrease the mortality rate and provide neuroprotection in infants with HIE [2–4]. However, the rates of death or disability in HIE infants treated with TH remain high [1]. Approximately 50% of newborns with moderate to severe HIE still die or suffer from long-term disabilities [1]. Many mechanisms have been proposed to explain the effects of TH, including the suppression of apoptosis, decreased inflammation, reduced levels of excitatory amino acids, reduced levels of reactive oxygen species, and a reduced cerebral metabolic rate [5, 6]. However, the cellular mechanism underlying the neuroprotective effect of TH is complex and has not yet been fully elucidated.

Astrocytes are the most abundant cells in the brain [7]. It is well established that astrocytes support neuronal function and survival via multiple mechanisms [8]. A key mediator of such paracrine neuroprotection is erythropoietin (EPO), particularly under hypoxic conditions [8]. Hypoxia-induced EPO expression is regulated at the transcriptional level by hypoxia-inducible factor (HIF) [9–12]. The EPO receptor (EPOR) provides a signaling mechanism for the neuroprotection effect of EPO in ischemic and neurodegenerative diseases [13, 14].

In the present study, we investigated whether hypothermia enhances the neuroprotective effect via EPO expression by astrocytes. To test this hypothesis, we examined the effect of hypothermia on EPO expression in cultured astrocytes. In addition, we investigated whether EPO released by astrocytes under hypothermic conditions attenuates neuronal damage.

## Methods

### Animals and reagents

The present study was approved by the Animal Care and Use Committee of Nagoya City University Graduate School of Pharmaceutical Sciences. All experiments were performed in accordance with institutional and U.S. National Institutes of Health guidelines for the care and use of laboratory animals. All rats were purchased from Japan SLC (Shizuoka, Japan). Recombinant EPO was purchased from R&D Systems (Minneapolis, MN, USA).

### Culture of primary astrocytes and neurons

Astrocytes were prepared from the cerebral cortex of postnatal day 1 Wistar rats, as described previously [15]. In brief, the cerebral cortex was dissected, trypsinized, and dissociated in low-glucose (1000 mg/dL) Dulbecco’s modified Eagle’s medium (DMEM; Wako, Osaka, Japan) supplemented with 10% fetal bovine serum (Bio sera), penicillin (80 units/mL), and streptomycin (0.2 mg/dL). After 7–10 days, the culture flasks were shaken overnight at 37 °C to remove cellular debris, microglia, oligodendrocytes, and their precursor cells. After shaking, cultured glial components were dislodged with 0.1% trypsin, and the cells were placed in 60-mm tissue culture dishes (1–1.5 × 10^6^ cells per dish). After secondary culture for 7–10 days, the medium was replaced with high-glucose (4500 mg/dL) DMEM (Wako) without fetal bovine serum for experiments in order to avoid effects of serum components and maintain cell viability. The cell population consisted of > 95% astrocytes, as determined by immunocytochemical staining with anti-glial fibrillary acidic protein antibody (DAKO, Glostrup, Denmark).

Primary neuron cultures were prepared and maintained as described previously [15]. In brief, neuron cultures were prepared from the cerebral cortex of embryonic day 17 Wistar rats. The cerebral cortex was treated as described above for primary astrocyte culture. Cells were dissociated on glass coverslips coated with BD Matrigel Basement Membrane Matrix Growth Factor Reduced (BD Bioscience, San Jose, CA, USA) in Neurobasal-A Medium (Invitrogen Carlsbad, CA, USA) containing 2 mM l-glutamine, 1% penicillin–streptomycin, and 2% B27 supplement (Invitrogen). Neurons were used for experiments after 7 days of culture. Immunostaining with microtubule-associated protein (MAP)2 antibody (Millipore, Billerica, MA, USA, Cat # MAB3418) revealed that neurons represented > 95% of the cell population.

### Hypoxia, glucose-deprivation, and hypothermia treatment of cultured cells

After the culture medium was replaced with fresh medium lacking fetal bovine serum, cells were cultured under oxygen/glucose deprivation (OGD). To induce hypoxia (1% O_2_), the culture plates were placed in an oxygen-controlled atmosphere culture incubator (WAKEN Biotech, Kyoto, Japan) at 37 °C. For glucose deprivation treatment, the culture medium was replaced with no-glucose DMEM (Wako). After 24 h of OGD, the culture plates were placed in a culture chamber at 37 °C or 33.5 °C for 24 h.

### Measurement of EPO production

During OGD and hypoxia alone and after hypothermia treatment of cultured cells, the conditioned medium was collected to measure EPO production using a commercially available ELISA kit (R&D Systems). Because the amount of EPO released into the culture medium was very low, the conditioned medium was concentrated using an Amicon Ultra-2 Centrifugal Filter Unit (Millipore) according to the manufacturer’s instructions.

### Quantitative reverse transcription–polymerase chain reaction (RT-PCR)

Quantitative RT-PCR analysis of selected genes was performed using a Thermal Cycler Dice Real-Time System (Takara Bio Inc., Otsu, Japan). Total RNA was isolated using RNA iso plus (Takara Bio Inc.) according to the manufacturer’s instructions. cDNA was generated from the total RNA samples via RT. RT was performed using PrimeScript RT Master Mix (Takara Bio Inc.), and the resulting cDNA was subjected to PCR-based amplification. Real-time PCR was performed using Go Taq (Promega Corp., Madison, WI, USA). Amplification was performed at 95 °C for 2 min, followed by amplification for 40 cycles at 95 °C for 15 s and 55 °C for 1 min. We normalized the relative quantification of target genes to endogenous β-actin mRNA abundance after confirming that the cDNAs from different genes were amplified with the same efficiency. The primer pairs used for amplification are shown in Table 1.

**Table 1:** Primer pairs used for polymerase chain reaction amplification

Gene	Primer sequence 5'→3'
β-actin	Forward: TCATGAAGTGTGACGTTGACATCCGT
	Reverse: CCTAGAAGCATTTGCGGTGCACGATG
EPO	Forward: TACCGTCCCAGATACCAAAGTCAAC
	Reverse: ACAGGCCTTGCCAAACTTCTACAG
HIF-1ɑ	Forward: CCAGAAGAACTTTTGGGCCG
	Reverse: TGTCCTGTGGTGACTTGTCC
HIF-2ɑ	Forward: ATCAGCTTCCTGCGAACACA
	Reverse: CAGCCTCGGCTTCAGATTCA

### Western blotting

To prepare nuclear extracts, cells were harvested and gently homogenized on ice using 10 mm Tris-HCl (pH 7.5) containing 1 mm EDTA, 0.5% Nonidet P-40, and a protease inhibitor cocktail (Sigma, St. Louis, MO, USA) for 10 min at 4 °C, as described previously [15]. Cell lysates were centrifuged at 20,000×*g* for 10 min at 4 °C to separate the cytoplasmic fraction as the supernatant. To collect nuclear extracts, insoluble material was dissolved in sodium dodecyl sulfate (SDS) sample buffer. Equal amounts of proteins were separated under denaturing conditions by electrophoresis on a 7.5% polyacrylamide gel containing SDS and electrotransferred onto polyvinylidene difluoride membranes (Immobilon-P; Millipore). The membranes were blocked with 5% skim milk in Tris-buffered saline containing 0.1% Tween 20 (TBS-T) at 4 °C overnight, followed by overnight incubation at 4 °C with primary antibodies against HIF-1α (1:300; R&D Systems, Cat # MAB1536) and HIF-2α (1:300; R&D Systems, Cat # AF2997) diluted in TBS-T. Blots were developed using the appropriate secondary antibody conjugated to horseradish peroxidase (1:5000; Cell Signaling Technology, Danvers, MA, USA, Cat # 7074 and 7076 and R&D Systems Cat # HAF109), and bands were visualized using an enhanced chemiluminescence method (Amersham Biosciences). To ensure antibody specificity in detecting proteins > 80 kDa in size, membranes were incubated with HIF-1α or HIF-2α only to prevent interference from strong nonspecific bands < 80 kDa in size. This technique enabled the identification of a specific band at ~ 120 kDa, which was not detected in astrocytes under normoxic conditions but appeared in astrocytes under hypoxic conditions. For normalization of protein loading, blots were stripped and reprobed with polyclonal anti-lamin B1 antibody (1:1000; Cell Signaling Technology, Cat # 12586) in TBS-T. Relative band intensities were determined by densitometry using ImageJ software (National Institutes of Health, Bethesda, MD, USA).

### Small interfering RNA (siRNA)

HIF-1α, HIF-2α, and negative control siRNAs were purchased from Sigma. Primary astrocytes were transfected with the indicated combinations of siRNA against HIF-1α or/and HIF-2α at a final concentration of 10 nM or negative control siRNA at a final concentration of 10 nM using LipofectAmine RNAiMAX transfection reagent (Invitrogen), according to the manufacturer’s recommendation. At 24 or 48 h after transfection, cells were exposed to hypoxic conditions for 12 or 24 h.

### Immunocytochemical staining

Cells were washed with PBS and then fixed in 3% paraformaldehyde in PBS at room temperature for 30 min. After washing, cells were blocked for 1 h at room temperature with blocking solution (3% bovine serum albumin and 0.1% glycine in PBS). The coverslips were incubated overnight at 4 °C with a rabbit polyclonal antibody against MAP2 (Millipore) and cleaved caspase 3 (Cell Signaling Technology, Cat # 9664). After washing, the coverslips were incubated for 1 h at room temperature with a secondary antibody conjugated with Alexa Fluor 488 or Alexa Fluor 594 (1:1000 dilution; Invitrogen, Cat # A11070 or A11020, respectively). After washing, sections were mounted on glass slides with ProLong Diamond anti-fade with 4′,6′-diamidino-2-phenylindole (Invitrogen). Stained cells were visualized using an LSM800 confocal microscope (Carl Zeiss, Germany).

### TdT-mediated dUTP nick-end labeling (TUNEL)

TUNEL staining was performed using a Takara In Situ Apoptosis Detection Kit (Takara Biomedicals, Tokyo, Japan), according to the manufacturer’s instructions. Cells were washed with PBS and then fixed in 3% paraformaldehyde in PBS at room temperature for 30 min. After washing and permeabilization, cells were incubated in labeling solution at 37 °C for 90 min, washed, and mounted on glass slides with ProLong Diamond anti-fade with 4′,6′-diamidino-2-phenylindole (Invitrogen). Stained cells were visualized using an LSM800 confocal microscope (Carl Zeiss).

### Blockade of EPO signaling

After OGD for 2 h, primary cultured neurons were further cultured in conditioned medium from astrocytes under normothermic or hypothermic conditions with control goat IgG (WAKO, Japan) or 1 μg/mL anti-EPO neutralization antibody (R&D Systems, Cat # AF959) for 24 h. After 24 h of incubation in conditioned medium, neurons were fixed and immunostained for determination of protein expression and TUNEL.

### Statistical analyses

All statistical analyses were performed using EZR (Saitama Medical Center, Jichi Medical University, Saitama, Japan), a graphical user interface for R software (The R Foundation for Statistical Computing, Vienna, Austria). More precisely, EZR is a modified version of R commander designed to add statistical functions frequently used in biostatistics [16]. Analysis of variance was used to compare differences in continuous data, followed by Bonferroni’s post hoc test. Pairwise comparisons were performed using the Student’s *t* test. Data are reported as the mean ± SEM. Statistical significance was set at *P* < 0.05.

## Results

### Expression of EPO in astrocytes cultured under OGD

**Fig. 1:**
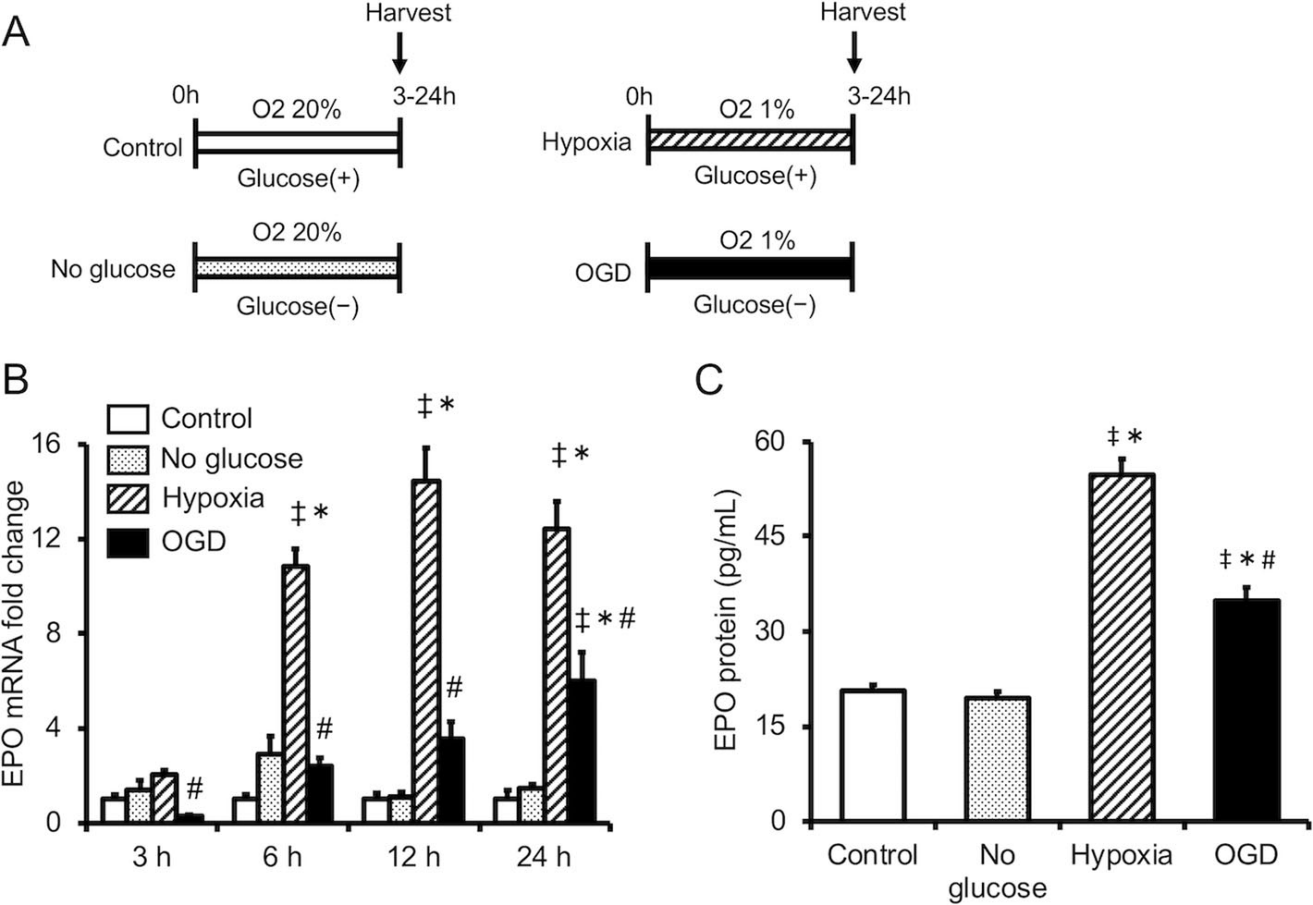
Change in expression of EPO in cultured astrocytes during hypoxia alone (hypoxia) or oxygen/glucose deprivation (OGD). **a** Time course of the change in EPO mRNA expression. Data are the mean ± SEM (*n*=3 in each group). **b** EPO protein concentrations were measured in astrocyte-conditioned medium 24 h after hypoxia and OGD. Data are the mean ± SEM (*n* =6 in each group). ^‡^*P* < 0.05 compared with the control group. ^*^P < 0.05 compared with the No glucose group. ^#^*P* < 0.05 compared with the Hypoxia group

Hypoxia reportedly increases EPO expression strongly [9]. Therefore, we first examined the time course of EPO gene expression in astrocytes cultured under conditions of hypoxia alone or OGD in order to determine whether OGD increases EPO expression similar to hypoxia alone. OGD induced EPO gene expression, although the expression level was significantly lower than that of cells cultured under hypoxia alone (Fig. 1a). The amount of EPO protein released into the culture medium was determined 24 h after culture under hypoxia alone or OGD. Under the OGD condition, the release of EPO from astrocytes into the culture medium was significantly increased compared with control cells and cells cultured with no glucose alone. However, OGD decreased EPO release compared with hypoxia alone (Fig. 1b).

### Expression of HIF-1α and HIF-2α in astrocytes cultured under hypoxia or OGD

**Fig. 2:**
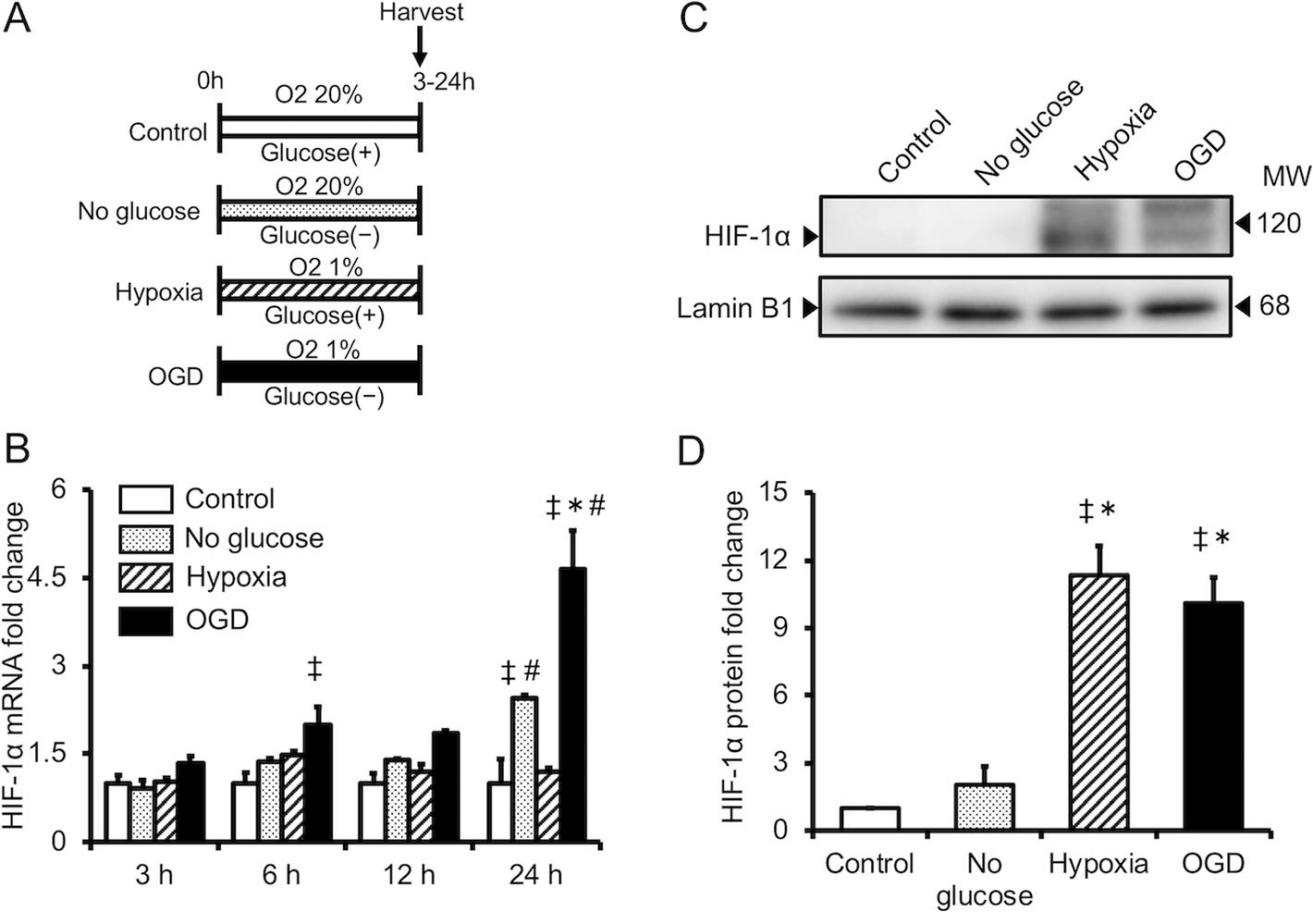
Effect of hypoxia alone (hpoxia) or oxygen/glucose deprivation (OGD) on HIF-1α expression in cultured astrocytes. **a** Quantitative RT-PCR analysis of HIF-1α mRNA levels during hypoxia or OGD. **b** Western blotting analysis of HIF-1α protein levels, and **c** semi-quantitative analysis of HIF-1α protein during hypoxia or OGD, as determined using ImageJ software. Data are the mean ± SEM (*n*=3 in each group). ^‡^*P* < 0.05 compared with the Control group. ^*^P < 0.05 compared with the No glucose group. ^#^*P* < 0.05 compared with the hypoxia group

**Fig. 3:**
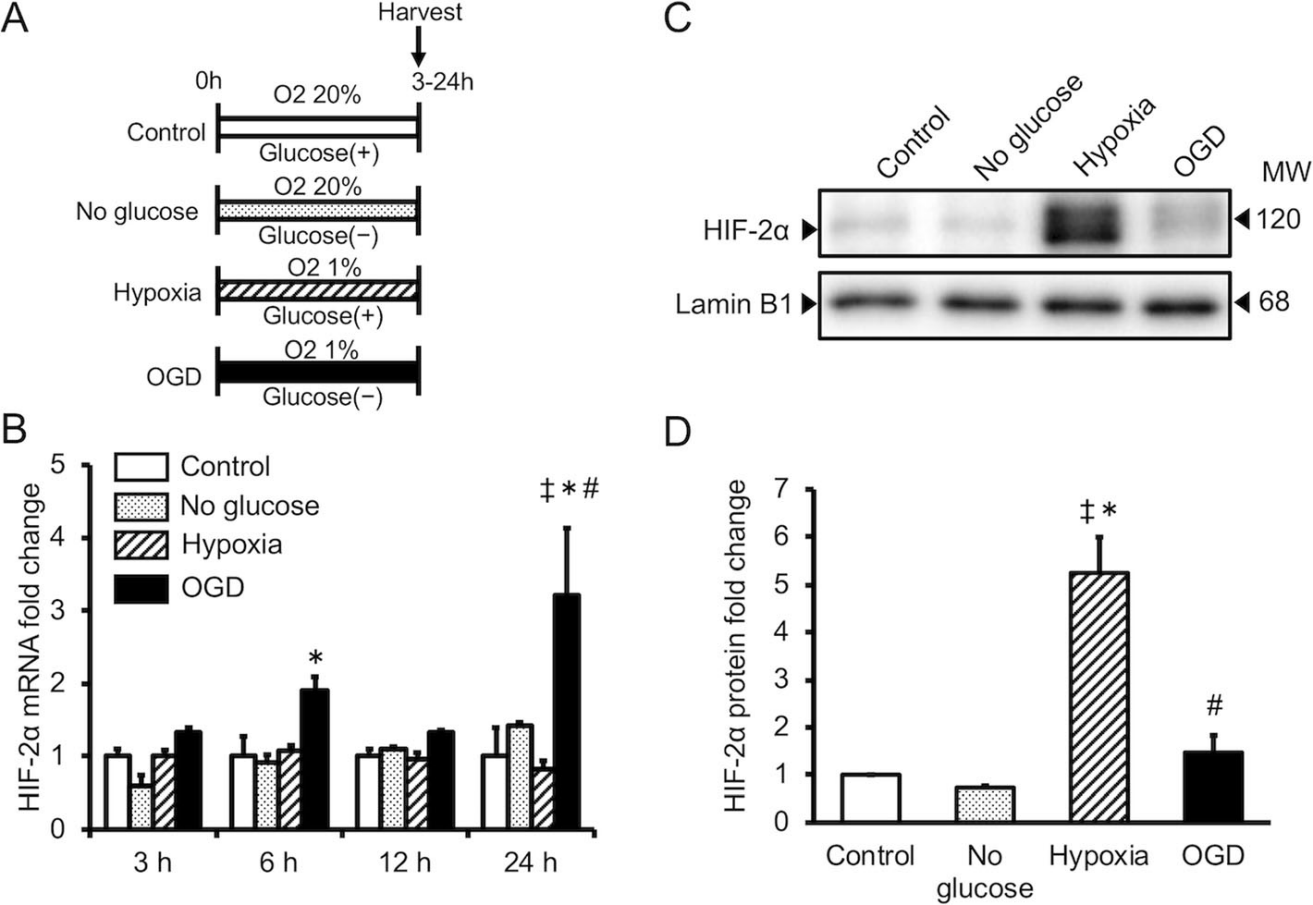
Effect of hypoxia alone (hypoxia) or oxygen/glucose deprivation (OGD) on HIF-2α expression in cultured astrocytes. **a** Quantitative RT-PCR analysis of HIF-2α mRNA levels during hypoxia or OGD. **b** Western blotting analysis of HIF-2α protein levels, and **c** semi-quantitative analysis of HIF-2α protein during hypoxia or OGD, as determined using ImageJ software. Data are the mean ± SEM (*n*=3 in each group). ^‡^*P* < 0.05 compared with the control group. ^*^P < 0.05 compared with the no glucose group. ^#^*P* < 0.05 compared with the hypoxia group

EPO expression is regulated by HIF proteins, which are transcription factors. The effect of OGD and hypoxia alone on HIF-1α and HIF-2α expression was evaluated in cultured astrocytes during hypoxia or OGD. OGD increased expression of the HIF-1α and HIF-2α genes, whereas hypoxia alone did not (Figs. 2a and 3a). HIF-1α and HIF-2α protein expression was increased in cells cultured under conditions of hypoxia alone and OGD, although cells cultured under OGD exhibited lower HIF-2α protein expression than cells cultured under hypoxia alone (Figs. 2b and 3b).

### Effect of HIF-1α and HIF-2α on EPO expression and neuronal protection in astrocytes

**Fig. 4:**
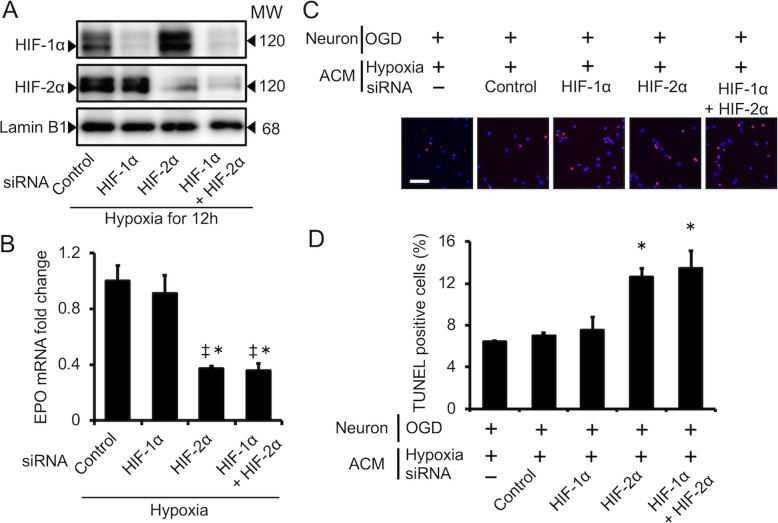
Effect of HIF-1α and HIF-2α on EPO expression and neuronal protection in astrocytes. **a** Astrocytes were transfected with siRNAs against HIF-1α and/or HIF-2α or negative control for 24–48 h prior to hypoxic stimulation. Western blotting analysis revealed HIF-1α and/or HIF-2α siRNA knockdown in astrocytes under hypoxia (12 ). **b** EPO mRNA in astrocytes transfected with siRNAs against HIF-1α and/or HIF-2α or negative control 24 h after hypoxia. Data are the mean ± SEM (*n*=3 in each group). ^‡^*P* < 0.05 compared with the Control siRNA group. ^*^P < 0.05 compared with the HIF-1α siRNA group. ^#^*P* < 0.05 compared with the HIF-2α siRNA group. **c** Knockdown of HIF-2α in astrocytes under hypoxic conditions increased the number of TdT-mediated dUTP nick-end labeling (TUNEL)-positive apoptotic neurons. Neurons were exposed to OGD for 2 h and reperfused in astrocyte-conditioned medium (ACM) of hypoxic astrocytes transfected with siRNAs against HIF-1α and/or HIF-2α or negative control for 48 h prior to hypoxic stimulation. Apoptotic neurons were identified using TUNEL staining. Nuclei were stained with 4,6-diamidino-2-phenylindole (DAPI; blue). Representative immunofluorescence images of TUNEL staining after 24 h of reperfusion. Bar, 50 μm. **d** Quantification of neuron apoptosis after OGD-associated injury. Knockdown of HIF-2α in astrocytes under hypoxic conditions promoted neuron apoptosis with 24 h of reperfusion. Data are the mean ± SEM (*n* =6 fields in each group). ^*^P < 0.05 compared with OGD-injured neurons in the hypoxic ACM group

Initially, we confirmed that HIF-1α and HIF-2α siRNAs suppressed HIF-1α and HIF-2α protein expression (Fig. 4a). We then examined whether specific knockdown of HIF-1α and/or HIF-2α suppresses EPO expression in astrocytes. Interestingly, HIF-2α knockdown significantly suppressed EPO expression in astrocytes under hypoxia, whereas HIF-1α knockdown did not (Fig. 4b). Moreover, HIF-2α knockdown significantly increased the number of TUNEL-positive apoptotic neurons in astrocyte-conditioned medium (ACM) under hypoxia, whereas HIF-1α knockdown did not (Fig. 4c, d).

### Expression of EPO in astrocytes cultured under hypothermia following OGD

**Fig. 5:**
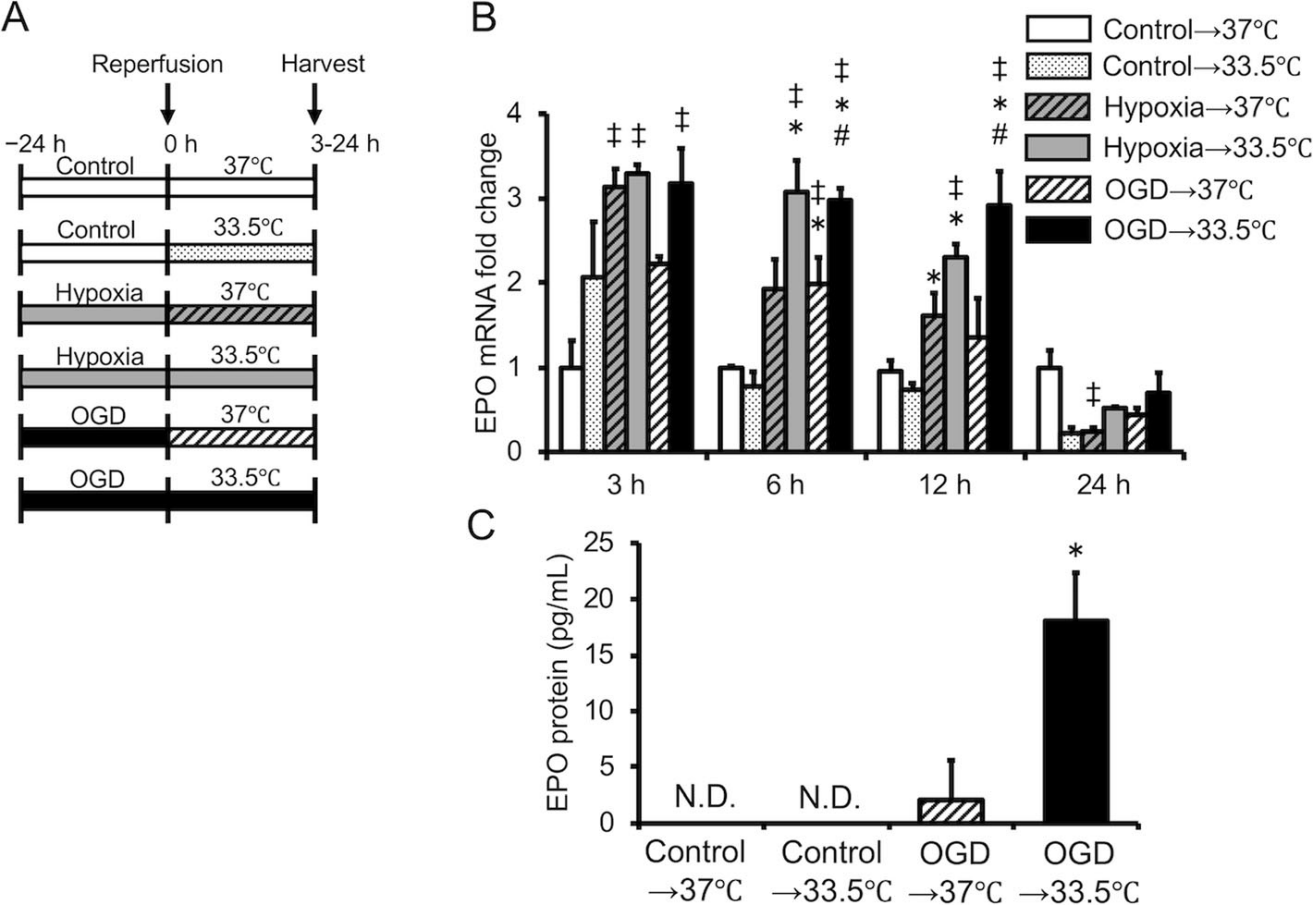
Change in expression of EPO in cultured astrocytes during hypothermia following oxygen/glucose deprivation (OGD). **a** Schematic representation of the experimental groups. **b** Time course of the change in expression of EPO mRNA. Data are the mean ± SEM (*n*=3 in each group). ^‡^*P* < 0.05 compared with the control→37 °C group. ^*^P < 0.05 compared with the control→33.5 °C group. ^#^*P* < 0.05 compared with the OGD→37 °C group. **c** Effect of hypothermia following OGD on EPO protein concentration in conditioned medium. EPO protein concentrations were measured in astrocyte-conditioned medium during 24 h of hypothermia, following the medium change after OGD. N.D., not detected. Data are the mean ± SEM (*n*=6 in each group). ^#^*P* < 0.05 compared with the OGD→37 °C group

The effect of hypothermia on EPO expression following OGD in cultured astrocytes was evaluated. We analyzed the time course of EPO gene expression in cells exposed to hypothermia following hypoxia alone or OGD (Fig. 5a). Expression of the EPO gene after hypoxia alone or OGD continued to be significantly higher in cells exposed to hypothermia compared with cells exposed to normothermic conditions until 12 h after hypoxia alone or OGD (Fig. 5b). EPO protein expression was significantly higher in cells subjected to hypothermia for 24 h following OGD than in cells exposed to normothermic conditions (*P* < 0.05; Fig. 5c).

### Expression of HIF-1α and HIF-2α in astrocytes cultured under hypothermia following OGD

**Fig. 6:**
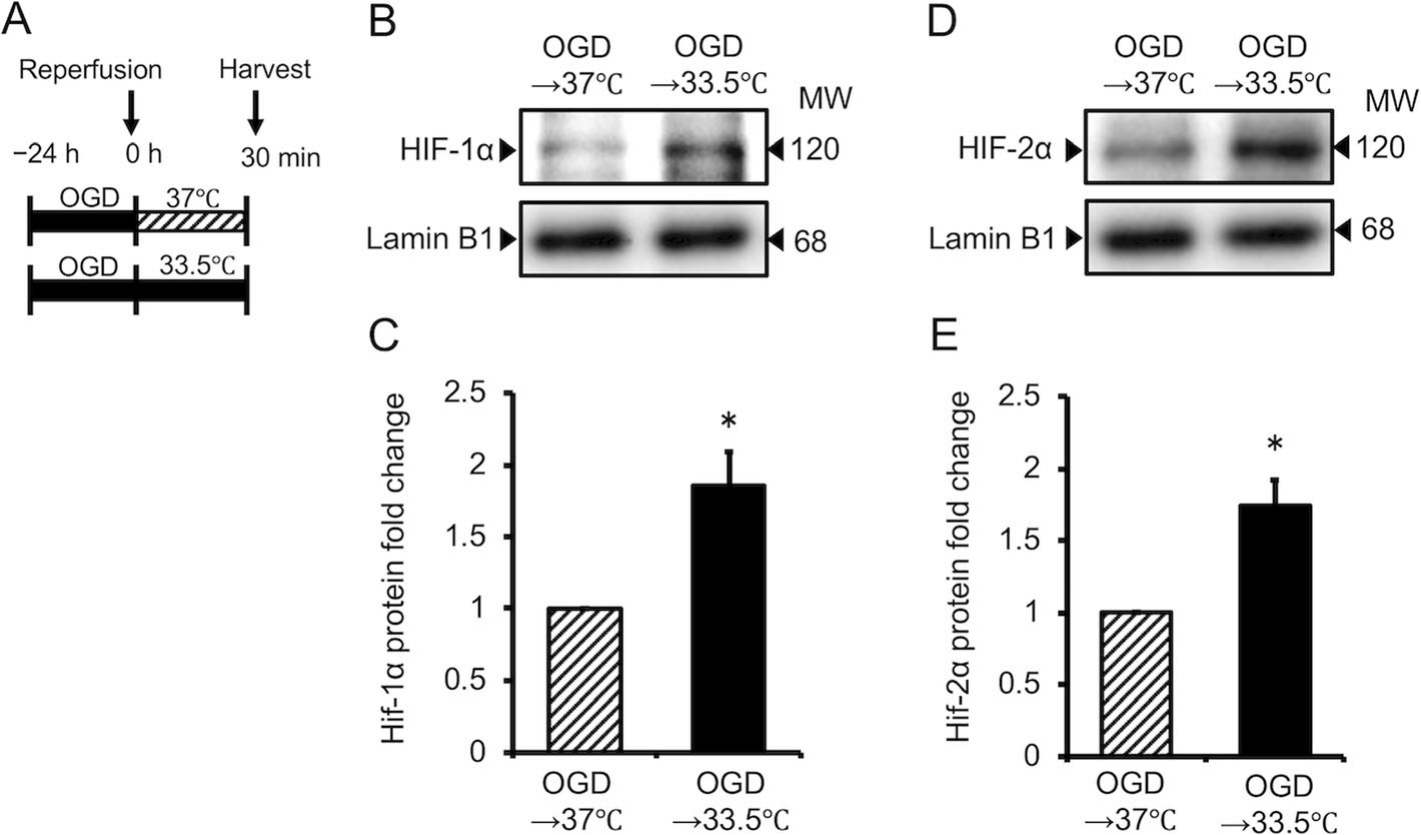
Effect of hypothermia after oxygen/glucose deprivation (OGD) on the expression of HIF-1α and HIF2α in cultured astrocytes 30 min after reperfusion. Western blotting analysis of HIF-1α (**a**) and HIF2α (**c**) protein levels, and semi-quantitative analysis of HIF-1α (**b**) and HIF-2α (**d**) protein, as determined using ImageJ software. Data are the mean ± SEM (*n*=3 in each group). ^#^*P* < 0.05 compared with the OGD→37 °C group

The effect of hypothermia on HIF-1α and HIF-2α protein expression in cultured astrocytes following OGD was evaluated. Expression of HIF-1α and HIF-2α protein in cells cultured under hypothermia for 24 h following OGD was significantly higher than that in cells cultured under normothermic conditions (*P* < 0.05 for both; Fig. 6).

### Neuroprotective effect of EPO secreted by astrocytes cultured under hypothermia

**Fig. 7:**
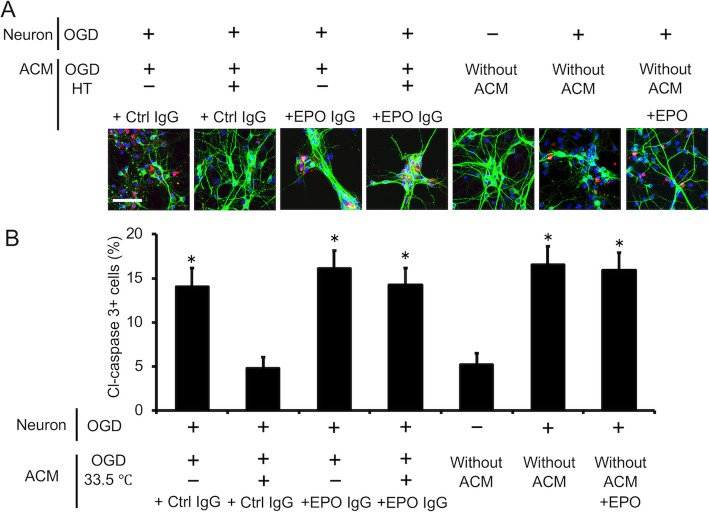
Endogenous EPO produced by astrocytes under hypothermic conditions reduced the number of cleaved caspase-3-positive apoptotic neurons. Neurons were exposed to oxygen/glucose deprivation (OGD) for 2 h and reperfused in astrocyte-conditioned medium (ACM) from OGD-hypothermic astrocytes with either control or EPO-neutralizing IgG. Neurons were stained with the neuron cell marker MAP-2 (green) and the apoptosis marker cleaved caspase-3 (red). Nuclei were stained with 4,6-diamidino-2-phenylindole (DAPI; blue). **a** Representative immunofluorescence images of MAP-2 staining after 24 h of reperfusion. Apoptotic cells were identified by positive staining for cleaved caspase-3. Bar, 50 μm. **b** Quantification of neuron apoptosis after OGD-associated injury. Blockade of EPO signaling using an EPO-neutralizing antibody promoted neuron apoptosis with 24 h of reperfusion. The addition of EPO to non-conditioned culture medium at the same concentration as in hypothermic ACM (18 pg/mL) did not change the population of cleaved caspase-3-positive cells. Data are the mean ± SEM (*n*=6 fields in each group). ^*^P < 0.05 compared with the hypothermic ACM/control IgG group

**Fig. 8:**
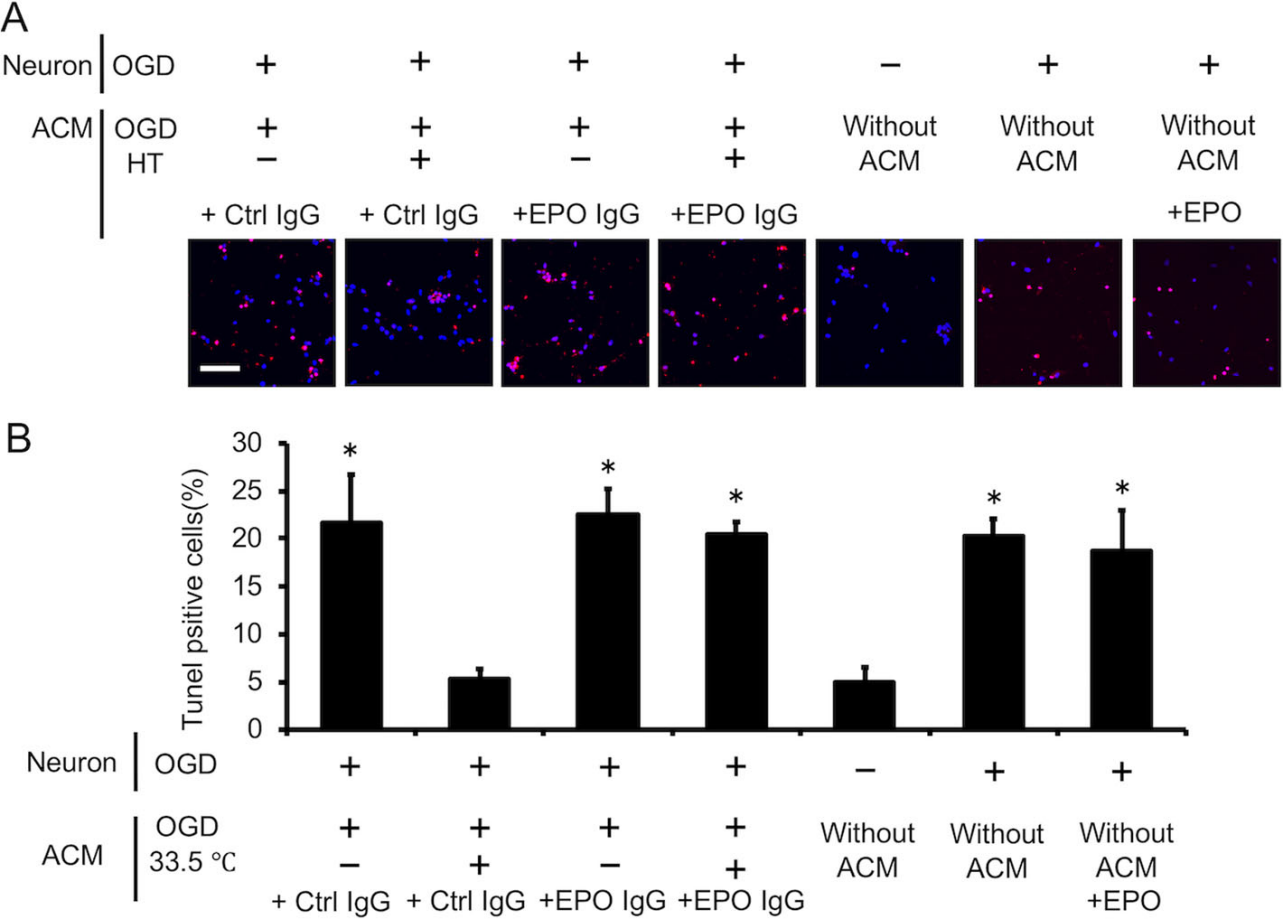
Endogenous EPO produced by astrocytes under hypothermic conditions reduced the number of TdT-mediated dUTP nick-end labeling (TUNEL)-positive apoptotic neurons. Neurons were exposed to OGD for 2 h and reperfused in astrocyte-conditioned medium (ACM) from OGD-hypothermic astrocytes with either control or EPO-neutralizing IgG. Apoptotic neurons were identified by TUNEL staining. Nuclei were stained with 4,6-diamidino-2-phenylindole (DAPI; blue). **a** Representative immunofluorescence images of TUNEL staining after 24 h of reperfusion. Bar, 50 μm. **b** Quantification of neuron apoptosis after OGD-associated injury. Blockade of EPO signaling using an EPO-neutralizing antibody promoted neuron apoptosis with 24 h of reperfusion. The addition of EPO to non-conditioned culture medium at the same concentration as in hypothermic ACM (18 pg/mL) did not change the population of TUNEL-positive cells. Data are the mean ± SEM (*n*=6 fields in each group). ^*^P < 0.05 compared with the hypothermic ACM/control IgG group

Under hypothermic conditions, astrocytes produced and secreted EPO into the medium (Fig. 5). We then examined whether increased astrocyte-derived EPO could attenuate neural cell damage under OGD (Additional file 1: Fig. S1). In the presence of ACM derived from astrocytes cultured under hypothermic conditions following OGD, the numbers of cleaved caspase-3- and TUNEL-positive apoptotic neurons were lower than in cells cultured in ACM from astrocytes cultured under normothermic conditions following OGD (*P* < 0.05 for both; Figs. 7 and 8). Interestingly, blockade of EPO signaling using an anti-EPO neutralizing antibody abrogated the anti-apoptotic effect of ACM from astrocytes cultured under hypothermic conditions following OGD (Figs. 7 and 8). Next, we examined whether exogenous EPO exerts a neuroprotective effect by adding the same concentration of EPO (18 pg/mL) as in hypothermic ACM to the neuron culture after OGD with non-conditioned culture medium. The addition of EPO to non-conditioned culture medium did not result in a neuroprotective effect compared with non-conditioned culture medium (Figs. 7 and 8). These data thus suggest that increased expression of EPO from hypothermic astrocytes suppresses neuron apoptosis induced by OGD-associated damage.

## Discussion

EPO, a hematopoietic cytokine, is produced primarily by interstitial fibroblasts in the adult kidney and by fetal hepatocytes [8]. The biological effects of EPO result from binding to the EPO-specific receptor, EPOR. Significant recent research interest has focused on the possible role of EPO as a novel neuroprotective agent [17]. In the present study, we found that EPO expression was consistently higher in astrocytes cultured under hypothermia following OGD compared with cells cultured under normothermic conditions. In addition, EPO expressed by astrocytes cultured under hypothermia following OGD attenuated neuronal damage.

Perinatal asphyxia with HIE is a leading cause of child mortality and long-term morbidities [1]. TH was recently established as the standard of care for infants diagnosed with moderate to severe HIE. TH is a promising neuroprotective intervention that has been shown to improve outcomes following nerve injury [6]. However, TH is only partially effective, with almost 50% of treated infants experiencing adverse outcomes [1–3]. The neuroprotective role of hypothermia has been well established in both experimental animals and patients with other brain injuries, including traumatic brain injury and stroke [18–20]. A number of mechanisms have been suggested to explain the effects of TH [5, 6]. For example, the neuroprotection associated with TH has been attributed to decreased metabolic rate, reduced generation of free radicals, amelioration of inflammation, and inhibition of excitotoxicity and apoptosis [5, 6]. However, the cellular mechanism underlying the neuroprotective effect of TH is complex and has yet to be fully elucidated.

Astrocytes are known to support neuronal function and survival via multiple mechanisms [8]. A key mediator of this paracrine neuroprotective effect is EPO, particularly under hypoxic conditions [8]. We reported previously that EPO secreted by astrocytes is protective for several brain cell types [21–23]. In addition, studies involving animal models have shown that EPO in the brain contributes significantly to neuroprotection [24–27]. Based on these data, we examined the effect of hypothermia on EPO expression in cultured astrocytes. We also investigated whether astrocyte-derived EPO suppresses neuronal damage during TH (Figs. 7 and 8).

Several studies demonstrated that hypoxia upregulates EPO expression via HIF, but there have been no reports describing the effect of OGD on EPO expression [9, 10]. Initially, we confirmed that OGD induced EPO expression similar to hypoxia alone. In subsequent experiments, we examined the effect of hypothermia on astrocytes subjected to OGD (but not hypoxia alone), because OGD better reflects HIE pathologically than does hypoxia alone. Interestingly, OGD elicited a less robust increase in EPO mRNA expression. The mechanism could involve destabilization of HIF-2α (but not HIF-1α) under OGD, although the precise mechanism of HIF-2α destabilization remains unclear. These data suggest that HIF-2α rather than HIF-1α predominantly regulates EPO expression and neural protective effects in astrocytes. Changes in the abundance of HIF-1α and HIF-2α mRNA during OGD might be compensation for HIF-2α destabilization. Indeed, knockdown of HIF-1α or HIF-2α increased levels of the other HIF-subtype mRNA (Additional file 2: Figs. S1 and S2). Under normoxic conditions, HIF-1α and HIF-2α protein stability is negatively regulated by ubiquitination and proteasome-dependent degradation [28]. HIF-2α, rather than HIF-1α, predominantly mediates the transcriptional activation of EPO expression under hypoxic conditions [8]. We previously reported that TNF-α suppresses only HIF-2α protein expression in hypoxic astrocytes, resulting in decreases in EPO mRNA and protein levels [15]. In the present study, hypothermia following OGD upregulated both EPO gene and protein expression. In addition, HIF-1α and HIF-2α protein expression was also upregulated. We also examined the effects of HIF-1α and HIF-2α on EPO expression and neural apoptosis using siRNA (Fig. 4). The results of these experiments suggest that EPO expression is predominantly upregulated due to increased HIF-2α protein expression and that hypothermia stabilizes HIF-EPO signaling in astrocytes. Hypothermia might decrease the prolyl hydroxylase reaction and/or subsequent steps in the proteasomal degradation pathway as a potential mechanism for hypothermic stabilization of HIF-1α and HIF-2α protein. Hypothermia might also affect regulation of EPO release from astrocytes via exosomes as another possible mechanism. Further studies using exosome release inhibitors will be needed to clarify the relationship between EPO release and hypothermia.

We also investigated whether EPO released from astrocytes inhibits the apoptosis of cultured neurons (Figs. 7 and 8). In the agreement with our previous report [22], culture in ACM under hypothermic conditions following OGD dramatically decreased the apoptosis of neurons subjected OGD in the presence of control IgG antibody. This effect was reduced in the presence of EPO-neutralizing antibody. In addition, we examined whether exogenous EPO exerts a neuroprotective effect. The addition of EPO at the concentration present in ACM did not have a neuroprotective effect, perhaps due to other neuroprotective components in ACM and/or differences between exogenous and endogenous EPO. Collectively, these data suggest that hypothermia attenuates neuronal damage via EPO released from astrocytes.

## Conclusion

In conclusion, hypothermia after OGD was found to stabilize HIF-EPO signaling in astrocytes. In addition, prolonged EPO production suppressed neuronal apoptosis induced by OGD-associated damage. Investigating the neuroprotective effect of EPO secreted by astrocytes under hypothermic conditions could thus contribute substantially to the development of novel neuroprotective therapies for HIE.

## Supplementary information

**Additional file 1: Figure S1.** (A) Schematic representation of the experimental groups in the present study. (B) Preparation of astrocyte-conditioned medium (ACM). (C) Preparation of neuron culture medium from ACM.

**Additional file 2: Figure S2.** Effects of HIF-1α siRNA and/or HIF-2α siRNA on HIF-1α and HIF-2α mRNA expressions in astrocytes. Astrocytes were transfected with siRNAs against HIF-1α and/or HIF-2α or negative control for 24-48 h prior to hypoxic stimulation. (A) HIF-1α mRNA in astrocytes transfected with siRNA against HIF-1α and/or HIF-2α or negative control 24 h after hypoxia. (B) HIF-2α mRNA in astrocytes transfected with siRNA against HIF-1α and/or HIF-2α or negative control 24 h after hypoxia. Data are the mean ± SEM (n=3 in each group). ^‡^*P* < 0.05 compared with the Control siRNA group. ^*^P < 0.05 compared with the HIF-1α siRNA group. ^#^*P* < 0.05 compared with the HIF-2α siRNA group.

## Data Availability

The datasets used and/or analyzed during the current study are available from the corresponding author on reasonable request.

## Abbreviations

ACM: Astrocyte-conditioned medium
DMEM: Dulbecco’s modified Eagle’s medium
EPO: Erythropoietin
EPOR: EPO receptor
HIF: Hypoxia-inducible factor
HIE: Hypoxic-ischemic encephalopathy
MAP-2: Microtubule-associated protein-2
OGD: Oxygen/glucose deprivation
RT-PCR: Reverse transcription–polymerase chain reaction
SDS: Sodium dodecyl sulfate
TUNEL: TdT-mediated dUTP nick-end labeling
