# Supplementary figures and images for: Prolonged astrocyte-derived erythropoietin expression attenuates neuronal damage under hypothermic conditions
Source: J Neuroinflammation. 2020 May 2;17:141. doi: 10.1186/s12974-020-01831-3 (PMC7195727; doi:10.1186/s12974-020-01831-3)

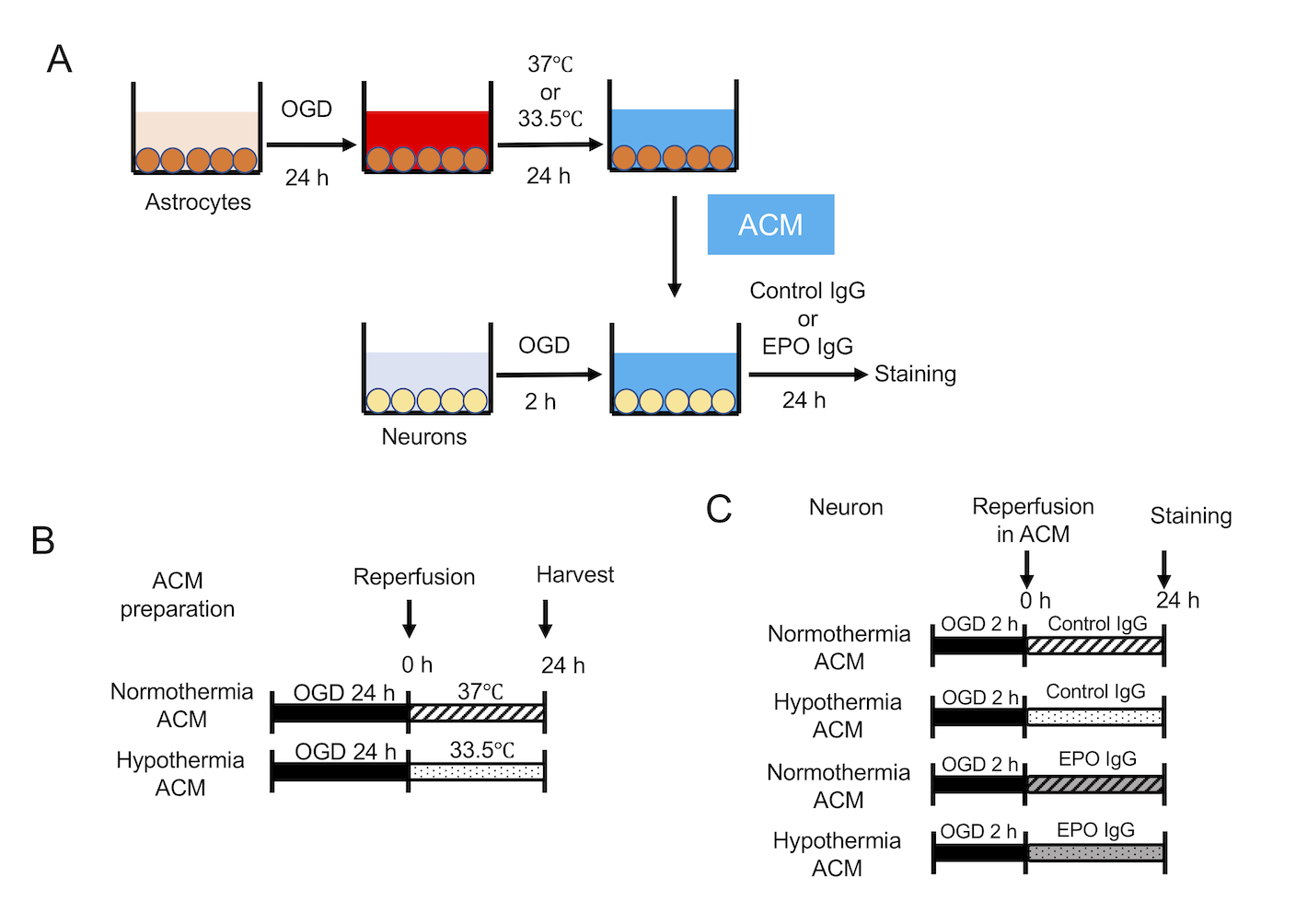

Supplement: Supplementary file 1 — Additional file 1: Figure S1. (A) Schematic representation of the experimental groups in the present study. (B) Preparation of astrocyte-conditioned medium (ACM). (C) Preparation of neuron culture medium from ACM. [file 12974_2020_1831_MOESM1_ESM.tiff]

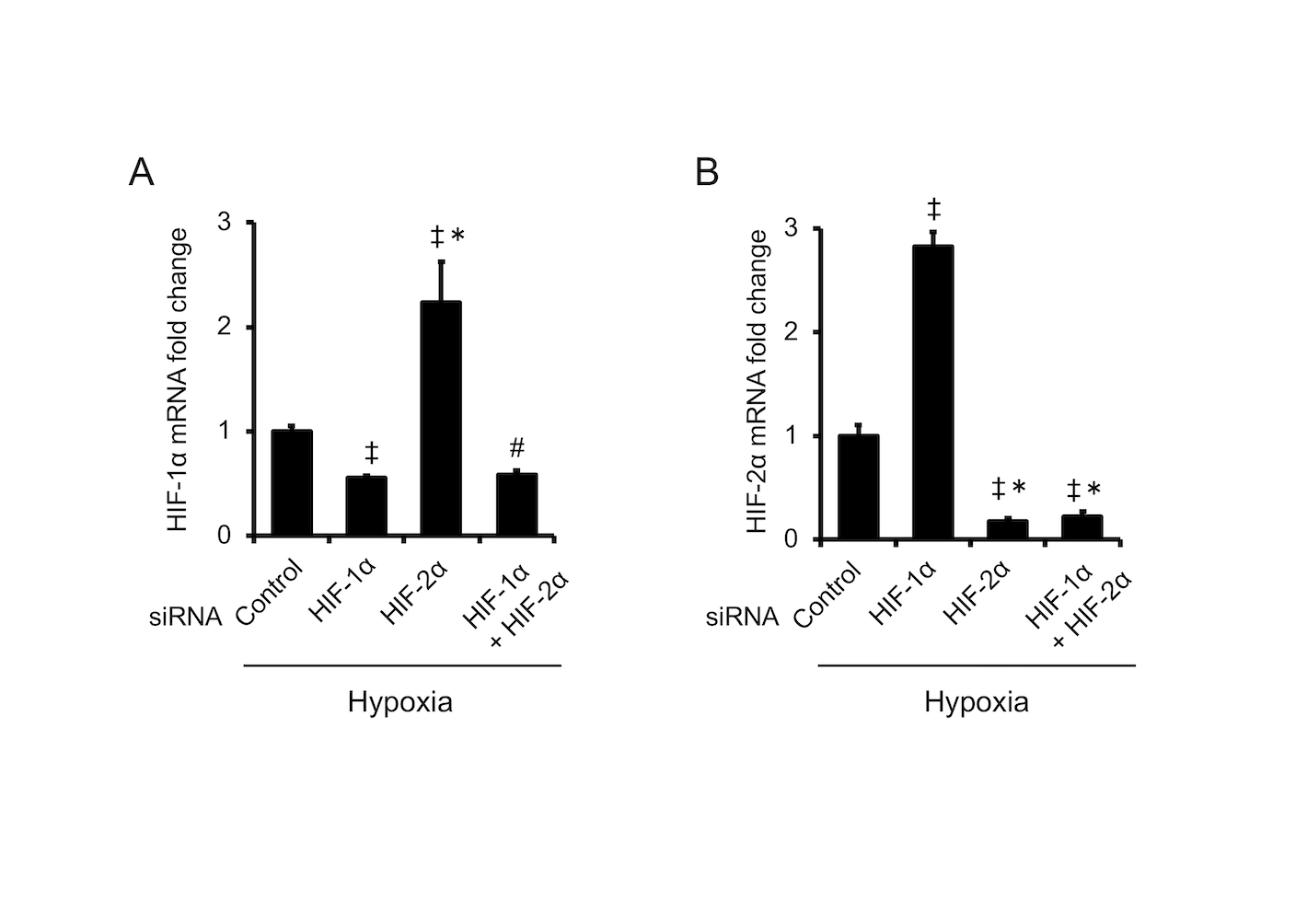

Supplement: Supplementary file 2 — Additional file 2: Figure S2. Effects of HIF-1α siRNA and/or HIF-2α siRNA on HIF-1α and HIF-2α mRNA expressions in astrocytes. Astrocytes were transfected with siRNAs against HIF-1α and/or HIF-2α or negative control for 24-48 h prior to hypoxic stimulation. (A) HIF-1α mRNA in astrocytes transfected with siRNA against HIF-1α and/or HIF-2α or negative control 24 h after hypoxia. (B) HIF-2α mRNA in astrocytes transfected with siRNA against HIF-1α and/or HIF-2α or negative control 24 h after hypoxia. Data are the mean ± SEM (n=3 in each group). ‡P < 0.05 compared with the Control siRNA group. *P < 0.05 compared with the HIF-1α siRNA group. #P < 0.05 compared with the HIF-2α siRNA group. [file 12974_2020_1831_MOESM2_ESM.tiff]
